# Nomogram predicting overall survival after surgical resection for retroperitoneal leiomyosarcoma patients

**DOI:** 10.3389/fendo.2023.1160817

**Published:** 2023-07-17

**Authors:** Aojia Zhuang, Xuetong Yue, Hanxing Tong, Yong Zhang, Fuchu He, Weiqi Lu

**Affiliations:** ^1^ Department of General Surgery, Institutes of Biomedical Sciences, Zhongshan Hospital, Fudan University, Shanghai, China; ^2^ State Key Laboratory of Proteomics, Beijing Proteome Research Center, National Center for Protein Sciences, Beijing, China

**Keywords:** nomogram, retroperitoneal leiomyosarcoma, survival, prediction of RLMS after surgery, FNCLCC

## Abstract

**Background:**

Surgery is the best way to cure the retroperitoneal leiomyosarcoma (RLMS), and there is currently no prediction model on RLMS after surgical resection. The objective of this study was to develop a nomogram to predict the overall survival (OS) of patients with RLMS after surgical resection.

**Methods:**

Patients who underwent surgical resection from September 2010 to December 2020 were included. The nomogram was constructed based on the COX regression model, and the discrimination was assessed using the concordance index. The predicted OS and actual OS were evaluated with the assistance of calibration plots.

**Results:**

118 patients were included. The median OS for all patients was 47.8 (95% confidence interval (CI), 35.9-59.7) months. Most tumor were completely resected (n=106, 89.8%). The proportions of French National Federation of Comprehensive Cancer Centres (FNCLCC) classification were equal as grade 1, grade 2, and grade 3 (31.4%, 30.5%, and 38.1%, respectively). The tumor diameter of 73.7% (n=85) patients was greater than 5 cm, the lesions of 23.7% (n=28) were multifocal, and 55.1% (n=65) patients had more than one organ resected. The OS nomogram was constructed based on the number of resected organs, tumor diameter, FNCLCC grade, and multifocal lesions. The concordance index of the nomogram was 0.779 (95% CI, 0.659-0.898), the predicted OS and actual OS were in good fitness in calibration curves.

**Conclusion:**

The nomogram prediction model established in this study is helpful for postoperative consultation and the selection of patients for clinical trial enrollment.

## Introduction

Leiomyosarcoma (LMS) is a mesenchymal malignant tumor with distinct smooth muscle differentiation (1), accounting for about 7-10% of all soft tissue sarcomas (STS). It is the second most common subtype of retroperitoneal sarcoma (RPS) (2). Currently, surgical resection is the best way to cure, but about 70% patients suffer from recurrence within five years after the surgical resection (3). The 5-year overall survival (OS) of the LMS patient is about 50%-60% after surgical resection for primary or recurrent disease (4, 5). The postoperative outcomes after extended resection for RPS have been reported, highlighting differences by specific histologic subtype (4). In addition, a report from the French Sarcoma Group pointed out that LMS represents a heterogeneous group of tumors, and retroperitoneal leiomyosarcoma (RLMS) shows totally different clinical outcomes and molecular features than others (6). Therefore, it is key to find good means to effectively incorporate our understanding of disease biology into clinical decision-making.

Nomogram is a statistical tool to predict the prognosis of an individual patient, and it has been widely applied in RPS (5, 7–9). But the distinct patterns of local and distant recurrence of the various subtypes of RPS suggest that separate nomograms for predicting the OS of RLMS patients will be useful (9). It has been reported that the tumor size, resection margin status, and tumor grade are risk factors for the poor prognosis of RLMS (10–12). However, no nomogram is constructed currently to predict the OS of RLMS patients only.

Therefore, this study aimed to investigate the independent prognostic factors for OS of RLMS patients after surgical resection and to construct a nomogram to predict the 1-, 2-, and 5-year OS of the RLMS patients.

## Methods

### Patients

118 consecutive patients who underwent surgical resection from September 2010 to December 2020 at General Surgery Department, Shanghai Public Health Clinical Center, Fudan University, Shanghai, China were included. The inclusion criteria were as follows: 1) histologically confirmed LMS, 2) tumors originating in the retroperitoneum, 3) complete clinical pathological information and follow-up information. LMS is diagnosed by pathological features stained by H&E, including oval or cigar-shaped nuclei with blunt ends, variable eosinophilic cytoplasm, and uniform positive staining for α-sma, desmin, and/or h-caldesmon (6). Gastrointestinal stromal tumors were excluded. The study was approved by the Ethics Committee of Shanghai Public Health Clinical Center. All enrolled patients signed informed consent for data collection during hospitalization.

The baseline characteristics including gender, age, metastatic disease, times of surgery, complete resection (complete: R0 and R1, negative gross margins; incomplete: R2, grossly positive margins), number of resected organ, tumor diameter, French National Federation of Comprehensive Cancer Centres (FNCLCC) grade, tumor node metastasis (TNM) stage, multifocality, inferior vena cava invasion, radiation, chemotherapy, and other therapy (focused ultrasound, interventional embolization and intraperitoneal hyperthermia chemotherapy). Treatment plans for all patients were determined by a multidisciplinary team of surgeons, medical oncologists, radiologists, and pathologists. Postoperative follow-up of the patients required physical examination and enhanced CT or MRI of the chest, abdomen and pelvic. The first follow-up was 3 months after surgery, then every 3 months for 2 years; 6 months for 5 years; and every 1 year after 5 years.

### Statistical analysis

The primary end-point of the study was OS (defined as the duration from date of the surgery to the date of death or follow-up) which estimated by the Kaplan–Meier method, and the differences between groups were compared with the log-rank test. Because the TNM staging system itself was a predictive tool, which already included relevant variables such as tumor size and tumor grade, it was not included in the risk factor analysis. Risk factors affecting survival were determined by COX regression. Variables with p-values <0.2 in univariate analysis were included in multivariate analysis. A nomogram was then constructed using the variables in the multivariate analysis with p-value < 0.1 to predict OS at 1, 2, and 5 years after surgery. The prediction accuracy of the nomogram was assessed by a calibration curve, and the c-index was judged the discriminative ability. Finally, the cohort was divided into a low-risk group and a high-risk group based on the values of the maximum sum of sensitivity and specificity (Youden index) (13). All the tests were two-tailed and p < 0.05 meant the difference was statistically significance.

Statistical analyses were performed with SPSS version 22.0 (Chicago, IL, USA) and R software (The R Foundation for Statistical Computing, Vienna, Austria; version 4.1.1; http://www.r-project.org/).

## Results

### Baseline characteristics

Characteristics of 118 patients were provided in **Table 1**. Most patients were female (n=99, 83.9%) without metastasis disease (n=106, 89.8%), and the tumor was completely resected (n=106, 89.8%). Patients with initial surgery, second surgery, and more than twice accounted for 39.8%(n=47), 34.7% (n=41), and 25.4% (n=30), respectively. The proportions of FNCLCC classification were equal as grade 1, grade 2, and grade 3, which were 31.4%, 30.5%, and 38.1%, respectively. The tumor diameter of 73.7% (n=85) patients was greater than 5 cm, the lesions of 23.7% (n=28) were multifocal, and 55.1% (n=65) patients had multiple organs resected. In terms of adjuvant therapy, 22 patients received radiotherapy, 46 received chemotherapy, and 15 received other therapies.

**Table 1:** Demographics and clinical characteristics of 118 patients with retroperitoneal leiomyosarcoma.

Characteristics	N (N=118)	% of Total
Gender		
Male	19	16.1
Female	99	83.9
Age, years		
<60	76	64.4
≥60	42	35.6
Metastatic disease		
Yes	12	10.2
No	106	89.8
Times of surgery		
Initial	47	39.8
Second	41	34.7
More than twice	30	25.4
Complete resection		
Yes	106	89.8
No	12	10.2
Number of resected organs		
0-1	53	44.9
>1	65	55.1
Tumor burden, cm		
≤5	31	26.3
>5	85	73.7
FNCLCC grade		
I	37	31.4
II	36	30.5
III	45	38.1
TNM stage		
I	35	29.6
II	16	13.5
III	55	46.6
IV	12	10.1
Multifocal disease		
Yes	28	23.7
No	90	76.3
Inferior vena cava invasion		
Yes	17	14.4
No	101	85.6
Radiation		
Yes	22	18.6
No	96	81.4
Chemotherapy		
Yse	46	39.0
No	72	61.0
Other therapy		
Yes	15	12.7
No	103	87.3

### Survival analysis

**Figure 1:**
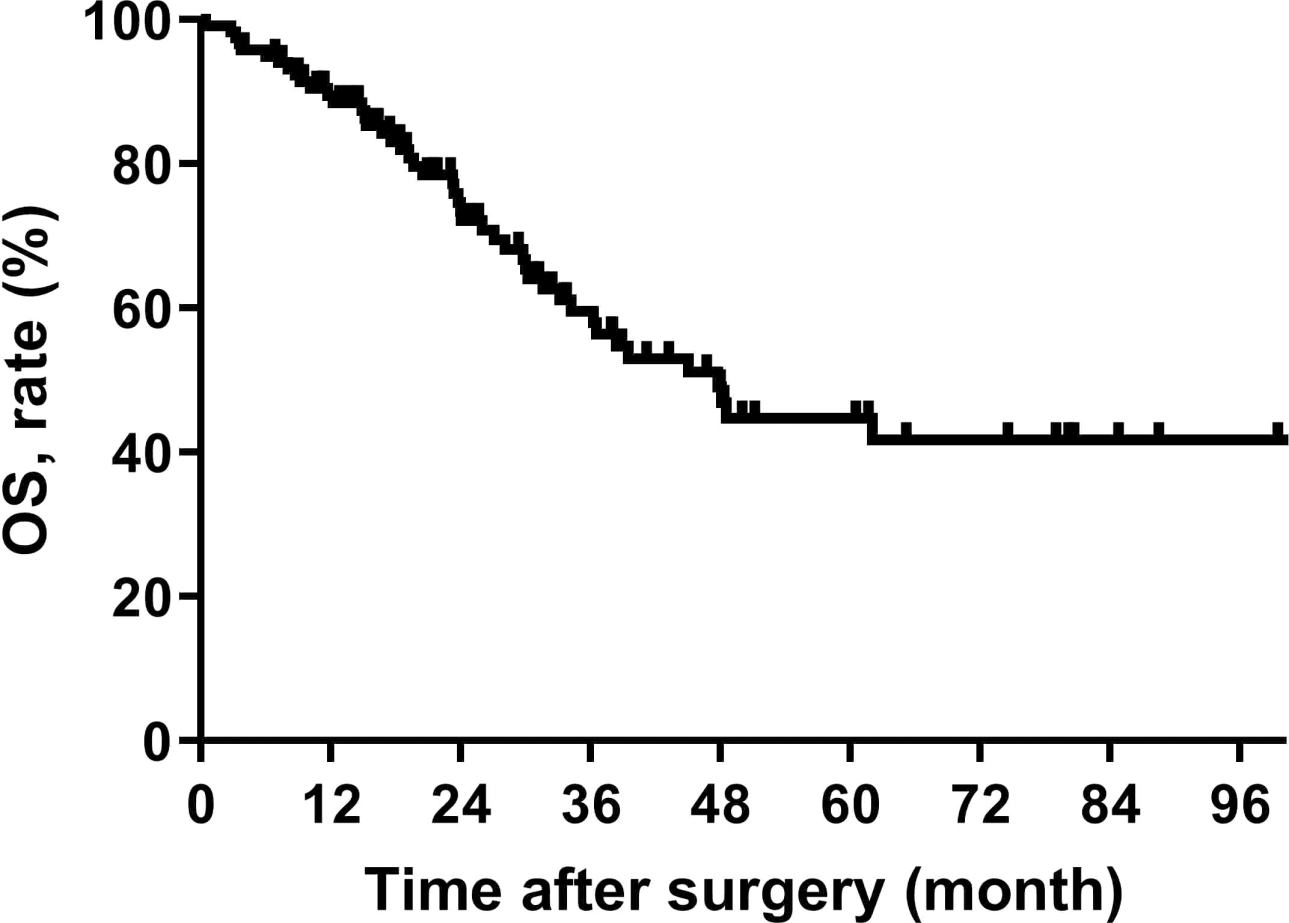
Overall survival in patients with retroperitoneal leiomyosarcoma.

The median OS for all patients was 47.8 (95% CI, 35.9-59.7) months, the median follow-up for all survivors was 31.0 (4 ~ 113 months) months, and the 5 years OS rate was 44.7% (95% CI, 32.6%-56.8%) (**Figure 1**).

The univariable analysis showed that the number of resected organs (p=0.012), tumor diameter (p=0.005), and FNCLCC grade (p<0.001) were related to OS. The results of multivariate analysis suggested that FNCLCC grade (p<0.001, Grade 2 vs. Grade 1 [HR=3.320, 95% CI 1.088-10.129], Grade 3 vs. Grade 1 [HR=11.693, 95% CI 4.183-32.689]) and multifocal lesions (p=0.025, HR=2.333, 95% CI 1.114-4.887) were independent risk factors of OS of patients (**Table 2**).

**Table 2:** Univariable and multivariable analyses to determine independent predictors of overall survival of retroperitoneal leiomyosarcoma.

Variables	Univariate analysis		Multivariate analysis	
	Hazard Ratio(95%CI)	P value	Hazard Ratio (95%CI)	P value
Gender female vs. male	1.006 (0.672-1.507)	0.975		
Age ≥60 vs. <60	1.242 (0.686-2.248)	0.475		
Metastatic disease yes vs. no	1.929 (0.814-4.570)	0.135	1.925 (0.740-5.-12)	0.180
Times of surgery		0.486		
second vs. first	0.664 (0.320-1.379)	0.272		
more than twice vs. first	1.020 (0.510-2.040)	0.955		
Complete resection no vs. yes	1.303 (0.846-2.007)	0.230		
Number of resected organ >1 vs. 0-1	2.173 (1.187-3.981)	0.012	1.797 (0.909-3.552)	0.092
Tumor burden >5 vs. ≤5	5.351 (1.655-17.299)	0.005	2.945 (0.861-10.070)	0.085
FNCLCC grade		<0.001		<0.001
II vs. I	3.352 (1.137-9.880)	0.028	3.320 (1.088-10.129)	0.035
III vs. I	9.355 (3.560-24.589)	<0.001	11.693 (4.183-32.689)	<0.001
Multifocal disease yes vs. no	1.906 (0.995-3.651)	0.052	2.333 (1.114-4.887)	0.025
Inferior vena cava invasion yes vs. no	1.259 (0.753-2.104)	0.380		
Radiation yes vs. no	1.767 (0.786-3.970)	0.168	1.400 (0.580-3.383)	0.454
Chemotherapy yes vs. no	1.005 (0.548-1.842)	0.987		
Other therapys yes vs. no	1.005 (0.395-2.555)	0.992		

### Nomogram development and validation

**Figure 2:**
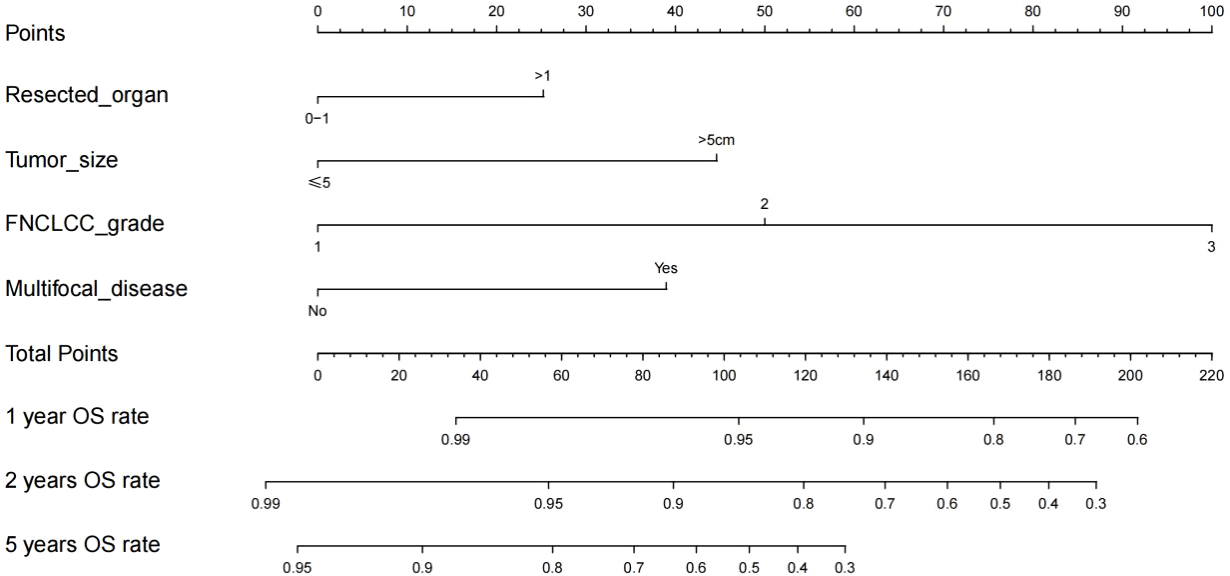
Nomogram for 1-year, 2-year and 5-year overall survival in patients with retroperitoneal leiomyosarcoma.

Variables with p<0.1 in multivariate analysis (the number of resected organs, tumor diameter, FNCLCC grade, and multifocal lesion) were referred to construct the nomogram to predict the OS of patients with RLMS who had the abdominal lesions resected (**Figure 2**).

**Figure 3:**
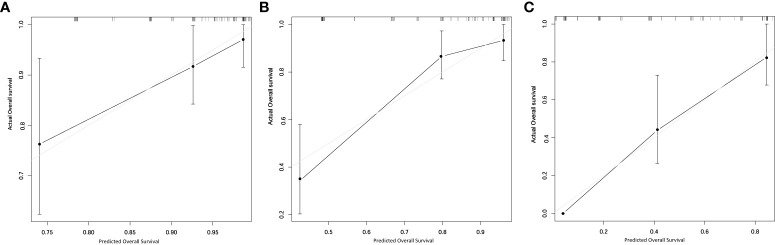
Calibration plots for internal validation of **(A)** 1-, **(B)** 2- and **(C)** 5-year overall survival nomogram.

The RLMS nomogram had an internal validation c-index of 0.779 (95% CI, 0.659–0.898), which was significantly better than the TNM staging system (c-index=0.697). And the calibration curve showed that the predicted value of OS was in good agreement with the actual value (**Figure 3**).

**Figure 4:**
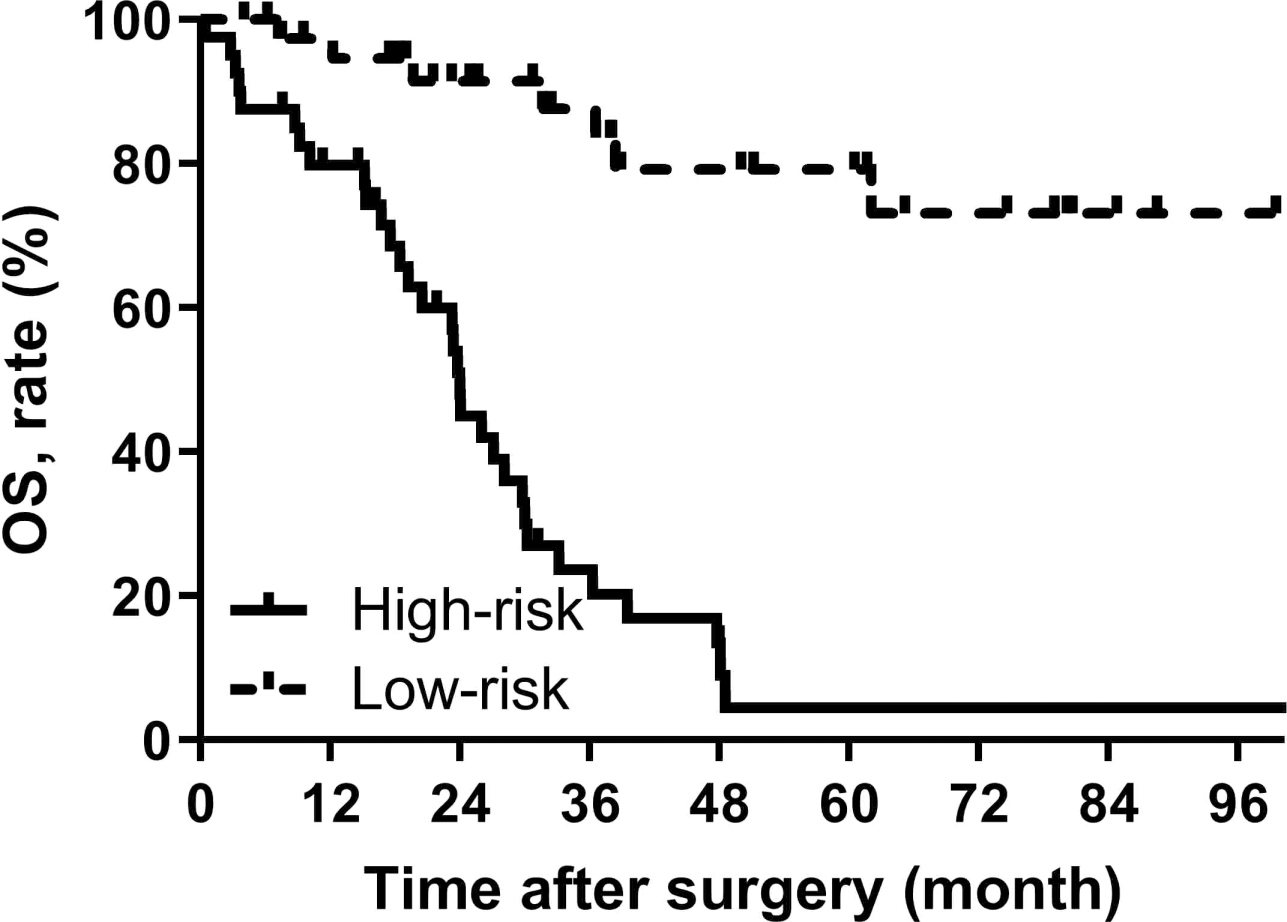
OS curves stratified by the score calculated by the nomogram and was stratified according to the risk score as follows: low-risk group (<100) and high-risk group (≥100).

The nomogram gave a specific value to quantify each variable. According to the total score, the Youden index was 0.572 with the cut off score of 126. 72 patients were included in the low-risk group (<126) and 46 were enrolled in the high-risk group (≥126), and their median OS was 116.5 (95% CI, NA) and 24.0 (95% CI, 22.9-25.0) months, respectively, showing a statistically significant difference (p<0.001) (**Figure 4**).

## Discussion

RLMS is a very rare mesenchymal malignant that originates in the retroperitoneal space. RLMS is fatal but rare disease, and metastasis, multifocal lesions and resection of multiple organs are usually contraindications to the surgical resection (14). Therefore, it is very important to analyze its long-term survival rate. In addition, the potential beneficial effects of surgical resection are not always obvious, resulting in very complicated decision-making. In other words, it is extremely difficult for clinically determine whether the patients with recurred RLMS could be treated by surgical resection. The nomogram established in this study is conductive for such decision or determination. For example, if the preoperative assessment shows no need for combined organ resection, the tumor diameter is smaller than 5 cm, FNCLCC shows grade 1 and single center disease, and even if it is a multiple recurrence disease, the postoperative 5-year OS rate may exceed 90%, so that patients have a high probability of benefiting from surgical resection. On the contrary, if the combined resection of multiple organs is needed, the tumor diameter is larger than 5cm, the FNCLCC showed grade 3 and multifocal disease, and the nomogram score was greater than 200 points, the postoperative 2-year OS is estimated to be less than 30%, so it has to be carefully considered to take surgical resection.

Since the prediction model can be simplified into a continuous numerical estimate tailored to individual patient conditions, the role of the nomogram prediction model has gradually become prominent with the help of precision medicine. Some nomogram prediction models have been incorporated into the staging system of the American Joint Committee on Cancer (AJCC) to improve the estimation accuracy of the prognosis for patients with subtype pathology. In the eighth edition of the AJCC manual, the nomogram by Trans-Atlantic Retroperitoneal Sarcoma Working Group for RPS (7) was included as a model that met all AJCC quality criteria. In this study, 523 patients were included to provide basis for establishing the nomogram prediction model, and 135 patients were used for foreign language validation. The c-index values for OS in the training set and validation set were 0.74 and 0.68, respectively. However, LMS only accounted for 17.6% in the training set, and its nomogram prediction model could only predict the OS of patients at the seventh year after the surgical resection, while the postoperative 5-year OS was only about 50%, showing limited practicality. In 2010, Anaya et al. established a nomogram model for predicting the postoperative 3-year and 5-year OS of patients by using the clinicopathological characteristics of 343 RPS patients, and its concordance index was 0.73. However, this model defined the pathological type of LMS as “others”, which was not listed separately (8). In 2019, Chandrajit et al. established a nomogram model for predicting the 6-year OS in RPS patients with first local recurrence based on the data of 602 patients from 22 sarcoma centers. Likewise, due to the rarity of RLMS, only 12.1% of LMS patients were included in the cohort (5).

In addition to nomogram prediction models for retroperitoneal sarcoma, there are also some LMS nomograms for the whole body. MingFeng et al. used the SEER database to establish a nomogram prediction model for predicting extremity LMS OS and cancer specific survival, with c-index of 0.776 and 0.835, respectively (15). And the LMS nomogram prediction model for the 5-year OS of urinary system was established by Oliver et al. with the c-index of 0.67 (16). Although the primary tumor site has a significant impact on the prognosis of LMS, there is currently no nomogram prediction model for LMS originating in the retroperitoneum. Therefore, this study developed and internally validated a novel RLMS-specific nomogram for predicting the 1-, 2-, and 5-year OS of patients with RLMS, and the c-index of the model was 0.779 (95% CI, 0.659-0.898). The calibration plots showed that the predicted OS rate was perfectly match with the actual value. In addition, to enhance the clinical utility, patients were further rolled into high-risk and low-risk groups based on the scores of the nomogram prediction model. The median OS of patients in the high-risk and low-risk groups was 116.5 (95% CI, NA) and 24.0 (95% CI, 22.9-25.0) months, respectively. The TNM staging system of the AJCC is the most commonly used method for staging STS, including RLMS. Staging is based on pathological findings, including tumor size, lymph node status, metastasis, and tumor grade based on the FNCLCC system (17). The RLMS nomogram in this study had a c-index of 0.779 (95% CI, 0.659-0.898) after internal validation, which was superior to the TNM staging system (c-index= 0.697). A more accurate prognosis could lead to a better postoperative counseling for patients, so it is possible to monitor the high-risk patients more appropriately.

In this study, the inclusion of radiation therapy in the multivariate analysis was based on recent reports highlighting its potential therapeutic benefits, despite having a p-value of 0.168 in the univariate analysis. The combination of radiation therapy with radical surgery has been shown to potentially be the standard of care for a subgroup of patients with RPS. Studies have demonstrated that patients treated with RT and surgery have significantly improved median overall survival and 5-year survival rates compared to those treated with surgery alone (p < 0.00001, p < 0.001). Additionally, median recurrence-free survival was significantly increased in patients treated with either preoperative or postoperative RT compared to those undergoing surgery alone (18). Another study further supports the use of radiotherapy in RPS, indicating that both preoperative and postoperative radiotherapy are significantly associated with improved overall survival compared to surgery alone (19). However, a recent randomized phase 3 study showed that the addition of preoperative radiotherapy did not improve local control rates or provide survival benefits, particularly in high-grade (G3) liposarcomas and LMS. Nevertheless, for recurrent retroperitoneal sarcomas primarily localized within the abdominal cavity, preoperative radiotherapy may help reduce the risk of local recurrence, especially in well-differentiated liposarcomas and low-grade dedifferentiated liposarcomas (20).Although radiation therapy did not demonstrate a significant association with overall survival in the further multivariate analysis of this study, considering the evidence from the mentioned high-quality studies, we still regard radiation therapy as a potential effective modality for treating leiomyosarcomas sarcoma of the retroperitoneum.

The results of survival analysis suggested that the FNCLCC grade and multifocal lesion were the independent risk factors for postoperative OS. The FNCLCC system is a commonly used histological grading system for STS, and it is one of the best indicators for predicting the metastasis-free survival and OS (21). This study was similar to a previous study reported by Qian Li et al., for RLMS patients with recurrent or metastatic disease who had a higher FNCLCC grade experienced worse prognosis (22). Consistent with previous reports on RPS, patients with multifocal lesions accounted for 23% of all subjects in this study; in addition, the mortality risk of patients with multifocal lesion increased by twice compared with non-multifocal lesion (23). Although there are some reports about the role of multifocal lesions in the prognosis of RPS, this study was the first to report it as an independent prognostic factor in RLMS.

There were some limitations for this study. First, this is a retrospective cohort study, and selection bias is inevitable. Second, the median follow-up time in this study was only 31 months, and further follow-up was needed to improve the reliability of the study. Thirdly, due to the complexity of treatments received by patients in our study cohort, there is a lack of information regarding adjuvant therapies. Finally, although the RLMS nomogram prediction model was internally validated, the cohort of this study was from an Asian tertiary hospital and was not externally validated, so its scope of use may be limited.

## Conclusions

We established an RLMS-specific nomogram prediction model that can accurately predict postoperative survival in RLMS patients. Dividing patients into high and low-risk groups by nomogram can assist doctors in clinical decision-making.

## Data availability statement

The original contributions presented in the study are included in the article/**Supplementary Material**. Further inquiries can be directed to the corresponding authors.

## Ethics statement

The studies involving human participants were reviewed and approved by the Ethics Committee of Shanghai Public Health Clinical Center (B2020-338). The patients/participants provided their written informed consent to participate in this study.

## Author contributions

AZ and HT developed the concept of the article. XY and AZ developed the design and methodology. AZ, XY,YZ, FH and WL contributed to the manuscript revision. AZ, XY and YZ contributed to the collection and analysis of clinical data. AZ, XY and HT contributed to the drafting of the manuscript. All authors contributed to the article and approved the submitted version.
