# Supplementary material for: Nomogram predicting overall survival after surgical resection for retroperitoneal leiomyosarcoma patients
Source: Front Endocrinol (Lausanne). 2023 Jul 17;14:1160817. doi: 10.3389/fendo.2023.1160817 (PMC10393052; doi:10.3389/fendo.2023.1160817)
Supplement: Supplementary file 1 [file Table_1.docx]

Supplementary Table 1 Demographics and clinical characteristics among the Initial, Second, and More than twice groups.

| Characteristics | Initial (% of Total) | Second (% of Total) | More than twice (% of Total) | P |
| --- | --- | --- | --- | --- |
| Gender |  |  |  | .892 |
| Male | 8 (17.0) | 7 (17.0) | 4 (13.3) |  |
| Female | 39 (83.0) | 34 (83.0) | 26 (86.7) |  |
| Age, years |  |  |  | 0.306 |
| <60 | 34 (65.9) | 29 (70.7) | 16 (53.3) |  |
| ≥60 | 16 (34.1) | 12 (29.3) | 14 (46.7) |  |
| Metastatic disease |  |  |  | 0.535 |
| Yes | 3 (6.3) | 5 (12.1) | 4 (13.3) |  |
| No | 44 (93.7) | 36 (87.9) | 26 (86.7) |  |
| Complete resection |  |  |  | 0.194 |
| Yes | 44 (93.7) | 34 (82.9) | 28 (93.3) |  |
| No | 3 (6.3) | 7 (17.1) | 2 (6.7) |  |
| Number of resected organs |  |  |  | 0.251 |
| 0-1 | 27 (57.4) | 22 (53.6) | 16 (53.3) |  |
| >1 | 20 (42.6) | 19 (46.4) | 14 (46.7) |  |
| Tumor burden, cm |  |  |  | 0.822 |
| ≤5 | 11 (23.4) | 12 (29.3) | 8(26.6) |  |
| >5 | 36 (76.6) | 29 (70.7) | 22 (73.4) |  |
| FNCLCC grade |  |  |  | 0.367 |
| I | 10 (21.3) | 15 (36.6) | 12 (40.0) |  |
| II | 16 (34.1) | 13 (31.7) | 7 (23.3) |  |
| III | 21 (44.6) | 13 (31.7) | 11 (36.7) |  |
| TNMG grade |  |  |  | 0.434 |
| I | 10 (21.3) | 14 (34.1) | 11 (36.7) |  |
| II | 6 (12.8) | 6 (14.6) | 4 (13.4) |  |
| III | 28 (59.6) | 16 (39.0) | 11 (36.7) |  |
| IV | 3 (6.3) | 5 (12.1) | 4 (13.4) |  |
| Multifocal disease |  |  |  | 0.185 |
| Yes | 7 (14.9) | 12 (29.3) | 9 (30.0) |  |
| No | 40 (85.1) | 29 (70.7) | 21 (70.0) |  |
| Inferior vena cava invasion |  |  |  | 0.130 |
| Yes | 9 (19.1) | 7 (17.0) | 1 (3.3) |  |
| No | 38 (80.1) | 34 (83.0) | 29 (96.7) |  |
| Radiation |  |  |  | 0.169 |
| Yes | 5 (10.7) | 9 (21.9) | 8 (26.6) |  |
| No | 42 (89.3) | 32 (78.1) | 22(73.4) |  |
| Chemotherapy |  |  |  | 0.047 |
| Yes | 16 (34.1) | 22 (53.6) | 8 (26.6) |  |
| No | 34 (65.9) | 19 (46.4) | 22(73.4) |  |
| Other therapies |  |  |  | 0.735 |
| Yes | 5 (10.6) | 5 (12.1) | 5 (16.6) |  |
| No | 42 (89.4) | 36 (87.9) | 25 (83.4) |  |
